# Retrospective Analysis of Cancer-Associated Myositis Patients over the Past 3 Decades in a Hungarian Myositis Cohort

**DOI:** 10.1007/s12253-019-00756-4

**Published:** 2019-10-23

**Authors:** Csilla András, Levente Bodoki, Melinda Nagy-Vincze, Zoltán Griger, Emese Csiki, Katalin Dankó

**Affiliations:** 1 Faculty of Medicine, Department of Medicine, Division of Oncology, University of Debrecen, Debrecen, Hungary; 2 Faculty of Medicine, Department of Medicine, Division of Rheumatology, University of Debrecen, Debrecen, Hungary; 3 Faculty of Medicine, Department of Medicine, Division of Clinical Immunology, University of Debrecen, Debrecen, Hungary

**Keywords:** Cancer-associated myositis (CAM), Dermatomyositis (DM), Polymyositis (PM), Cancer, Muscle weakness

## Abstract

Association between cancer and myositis has been extensively reported and malignancy is a potentially life-threating complication in myositis. In this retrospective study authors give an overview of Hungarian cancer-associated myositis (CAM) patients treated at a single centre managing 450 myositis patients. All patients were diagnosed according to Bohan and Peter. Statistical analysis of disease onset, age, sex, muscle, skin and extramuscular symptoms, muscle enzymes, presence of antibodies, treatment and prognosis was performed. 43 patients could be considered as having CAM. 83.72% had cancer within one year of diagnosis of myositis. Most common localizations were ductal carcinoma of breast and adenocarcinoma of lung. Significant differences were observed between CAM and the non-CAM control patients: DM:PM ratio was 2.31:1 vs. 0.87:1, respectively (*p* = 0.029), age at diagnosis was 56.60 ± 12.79 vs. 38.88 ± 10.88 years, respectively (*p* < 0.001). Tumour-treatment was the following: surgical removal in 55.81%, chemotherapy in 51.1%, radiotherapy in 39.53%, hormone treatment in 18.6%, combination therapy in 51.16% of patients. Muscle enzyme levels of patients undergoing surgery were significantly reduced after intervention. 36 patients died (83.72%); 25 DM (83.33%) and 11 PM patients (84.62%); 5 years survival was 15.4% for PM and 27.5% for DM. This study demonstrates that DM, distal muscle weakness, asymmetric Raynaud’s phenomenon, older age, ANA-negativity are risk factors for developing malignancy and polymyositis patients have less chance of long-lasting survival. It is very important to think about cancer and follow every single myositis patient in the clinical routine because survival rate of CAM is very poor.

## Introduction

Idiopathic inflammatory myopathies (IIMs) are systemic, chronic, autoimmune diseases, characterised by symmetrical, proximal muscle weakness. The IIMs fall into six clinico-pathological categories: (1) dermatomyositis (DM; juvenile, adult), (2) polymyositis (PM), (3) overlap syndromes (OM), (4) cancer-associated myositis (CAM), (5) inclusion body myositis (IBM), and (6) focal and diffuse myositis, including necrotizing autoimmune myopathy (NAM) [1–11]. The link between myositis and cancer was originally noticed in the early 1900’s [12], also by Bohan and Peter in their classification in 1975 [13], and has been than proved by large population-based studies [14]. Modern epidemiologic works have provided strong support for this association and evidence that it may also be true for PM, but the risk is lower than that for DM patients [15]. This well-recognised association between IIM and malignancy is a remarkable complication contributing to increased mortality in myositis. While the exact pathogenesis of CAM remains unclear, a paraneoplastic nature was assumed in the majority of these patients because cancer diagnosis and myositis onset seemed to temporally coincide [16]. The discovery of myositis-associated antibodies (MAAs) and myositis-specific antibodies (MSAs) led to the development of the clinico-serological classification and the recently discovered anti-TIF1gamma antibodies seem to be strong related to malignancy [17, 18]. The recent publication of the European League Against Rheumatism and American College of Rheumatology (EULAR/ACR) classification criteria for IIM [19, 20] was a big step forward because it makes the everyday work of the physician easier. Despite its many advantages this classification criteria does not contain any specific factor that can help in the recognition of CAM patients. There is limited evidence for specific treatment strategies in IIM and this also applies for CAM [21]. It is still of primary importance to diagnose these complicated cases as early as possible because the possibility of survival increases with early identification and therapy.

## Aims

In this retrospective study authors aimed to give a retrospective overview of Hungarian myositis patients with malignancy treated at a single centre. At the Division of Clinical Immunology, Department of Medicine, University of Debrecen, Hungary, 450 myositis patients have been treated since the end of the 1980’s, 304 patients with PM, OM, IBM or NAM and 146 patients with DM. Our aim was to study the clinical, immunological and therapeutic characteristics of myositis cases associated with cancer from the last 3 decades.

## Methods

### Data Collection

We retrospectively collected data from the University’s computerized patient record system, called eMedSolution. All selected myositis patients had a definitive or probable diagnosis for myositis (muscle weakness, high muscle enzyme levels, plus positive EMG and / or positive muscle biopsy in polymyositis or skin symptoms in dermatomyositis, according to Bohan and Peter). No juvenile DM/PM cases were selected. All myositis patients that had cancer during their life were primarily enrolled to the study. Based on these, 60 cases could be identified. Analysis of following epidemiologic, clinical, laboratory and therapeutic data were performed: age, sex, muscle symptoms, skin symptoms, extramuscular symptoms, serum level of creatine kinase, lactate dehydrogenase, presence or absence of MSAs or MAAs, drugs used for the treatment of cancer and myositis, and prognosis of patients.

### Detection of Antibodies

Immunoserological analyses, which were performed at the Department of Laboratory Medicine, University of Debrecen, included tests for the following autoantibodies. Antinuclear antibodies (ANA) were determined by indirect immunofluorescence on HEp-2 cells (Viro-Immun Labordiagnostika GmbH, Oberursel, Deutschland); ANA positivity was assessed at 1:40 dilution. Anti-Scl70, anti-Sm/RNP were determined in all patients by ELISA (Hycor Biomedical Inc., Garden Grove, CA, USA). Anti-Jo-1, anti-Mi-2, anti-Pm-Scl, anti-Ku antibodies were detected by membrane-fixed immuno-blot (Orgentec Diagnostika GmbH, Mainz, Deutschland). Anti-SSA and anti-SSB were determined by ELISA (Hycor Biomedical Inc., Garden Grove, CA, USA), as well as anti-U1RNP (Orgentec Diagnostica GmbH, Mainz, Deutschland). These commercially available methods were used following the manufacturer’s protocol.

### Diagnosis of Clinical Parameters, Muscular and Internal Organ Involvement

EMG was performed using the Buchtal-method at Department of Neurology, University of Debrecen. Muscle biopsies were performed by surgeons and analysed by a neuropathologist. Pulmonary fibrosis was defined as present by radiographic findings and pulmonary function tests (spirometry, DLCO). Dysphagia was diagnosed by barium radiography of the oesophagus. Cardiac involvement was encoded in case of pericarditis, myocarditis, conduction disturbances, myocardial ischemia and recurrent arrhythmia; it was assessed by ECG, two-dimensional and Doppler echocardiography. Imaging studies (US, X-ray, CT, HRCT, MRI, PET-scan) were done, if needed, at the Department of Radiology, University of Debrecen.

### Statistics

To compare groups with categorical data Pearson Chi-square (χ^2^) test was used. To compare groups with small number of cases Fisher’s exact test was used. Statistical analysis was made using SPSS 20.0 statistical software. When creating a small group of patients as control group random number generator had been used. During statistical analysis, the *P* value less than 0.05 was regarded as statistically significant.

## Results

**Fig. 1:**
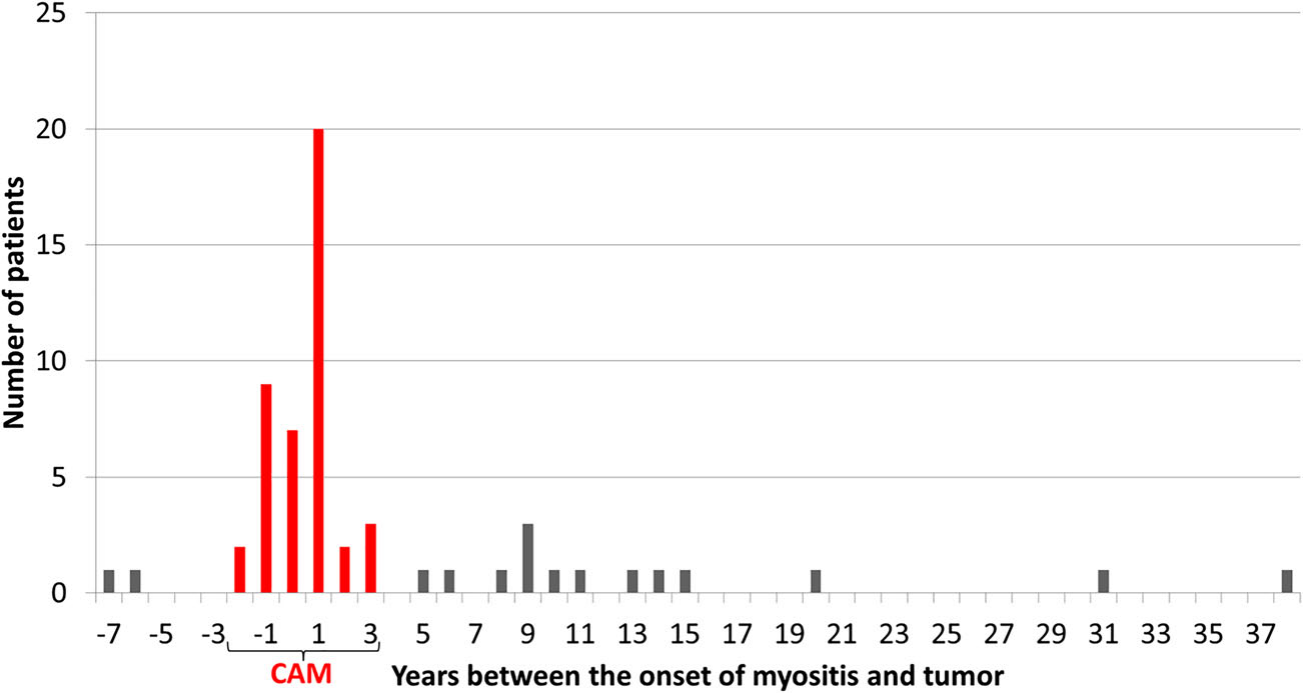
43 patients had CAM of the myositis population

There are no clear literature data on when to consider a case as a CAM patient because the exact characteristics of the timely association of malignant disease and myositis are not known. Based on the previous work of our group [22] plus relying on data from other population studies [23], we evaluated the myositis as „tumour associated” in the following cases: 1. if the tumour was diagnosed within two years before muscle symptoms; 2. if the cancer process has been diagnosed within three years after the onset of myositis symptoms. Accordingly, out of a total of 60 patients who have ever had cancer, a total of 43 patients could be considered as having CAM (Fig. 1.). Hereinafter we deal with these 43 patients. In seven patients symptoms of myositis and cancer appeared simultaneously (±3 months); in another 29 patients cancer was diagnosed ±1 year of myositis onset. This means that 83.72% of all patients had cancer within one year of the diagnosis of myositis. DM:PM ratio was 2.31:1, with 30 DM and 13 PM patients. Age at the diagnosis of CAM was 56.60 ± 12.79 (55.9 ± 13.71 for DM and 58.23 ± 10.71 for PM patients). Female:male ratio was 2.07:1, with 29 women and 14 men, for DM patients 2.33:1 and for PM patients 1.6:1.

In our opinion it is important to know when these cases were diagnosed. If we divided the 29 years between 1990 and 2018 into 5-year periods, the number of patients in each time interval was as follows: 1990–1994: one patient, 1995–1999: six, 2000–2004: twelve, 2005–2009: thirteen, 2010–2014: nine, 2015-nowadays: two patients.

One of the most important questions is the type of cancers that were associated to the myositis. These data can be followed on Table 1. The two most common anatomical localizations were breast and lung while the most common histological types were ductal carcinoma of the breast and adenocarcinoma in different localizations.

**Table 1:** Types of malignancy in our CAM patients

Number of patients	Localisation of cancer	Type of cancer
15	breast	ductal carcinoma (14) lobular invasive carcinoma (1)
12	lungs	adenocarcinoma (4) squamous cell carcinoma (2) small-cell carcinoma (2) not known (4)
3	mouth cavity	squamous cell carcinoma of the epipharynx (2) mucoepidermoid carcinoma of the salivary glands (1)
2	colon	adenocarcinoma
2	ovary	cystadenocarcinoma
2	hematological malignancy	lymphoma (1) multiple myeloma (1)
1	stomach	adenocarcinoma
1	pancreas	adenocarcinoma
1	urinary bladder	carcinoma transitiocellulare
1	penis	squamous cell carcinoma
1	prostate	adenocarcinoma
1	brain	ependymoma
1	cervix uteri	cystadenocarcinoma

To compare some of the most important characteristics of CAM patients and “simple” myositis patients we generated a control group. Between 1990 and 2018, we also created six “5-year intervals”. Out of the 390 non-CAM patients, 43 patients were randomly selected using a computer (random number generator); there were as many patients in each interval as it was by the CAM group, representing the whole non-cancer associated myositis population. In this group, no patient had ever malignancy. The DM:PM ratio was 0.87:1. Age at diagnosis of myositis was 38.88 ± 10.88, female:male ratio was 2.31:1 with 30 women and 13 men. These data are summarized in Table 2. We can see that there was a significant difference between the two groups in the case of the DM:PM ratio and the disease onset. Most important clinical, serological and therapeutic data of these two groups are summarized and compared in Table 3.

**Table 2:** Important demographic data of CAM patients and “non-malignancy” patients

	CAM patients	Myositis patients with no malignancy	*P* value
Number of patients	43	43	
DM:PM ratio	2.31:1	0.87:1	**0.029**
Age at disease onset	56.6 ± 12.79	38.88 ± 10.88	**0.001**
Female:male ratio	2.07:1	2.33:1	0.816

**Table 3:** Comparison of CAM and non-cancer myositis patients

	CAM patients (*n* = 43)	Myositis patients with no malignancy (n = 43)	P value
Skin symptoms			
Proximal muscle weakness	43	43	1
Distal muscle weakness	7	0	**0.012**
Gottron’s papules	24	14	**0.03**
Gottron’s sign	15	11	0.348
Heliotrop rash	18	15	0.506
V-sign	22	10	**0.007**
Periungual teleangiectasia	10	4	0.08
Mechanics’ hand	5	5	1
Other clinical signs			
Raynaud’s phenomenon	7	15	**0.048**
Arthralgia/arthritis	20	29	0.05
Fever at disease onset	2	0	0.494
Interstitial lung disease	6	7	0.763
Dysphagia	17	20	0.514
Heart involvement	3	3	1
Autoantibodies			
ANF	12	28	**0.001**
Anti-Mi-2	2	9	**0.024**
Anti-SRP	3	1	0.616
Anti-Jo-1	3	0	0.241
Anti-PL-7	0	0	–
Anti-PL-12	0	0	–
Anti-SSA	5	5	1
Anti-SSB	2	2	1
Anti-Sm-RNP	2	2	1
Anti-PM-Scl	0	1	1
Anti-Ku	1	0	1
Anti-U1RNP	0	0	–
Anti-Scl-70	0	1	1
Therapy			
Steroids	43	42	1
Methotrexate	5	15	**0.011**
Azathioprine	5	15	**0.011**
Cyclophosphamide	4	10	0.08
Cyclosporine A	4	18	**0.001**
IVIG	2	7	0.156

Some notable results of symptoms in the CAM group were for example the relative high number of distal muscle weakness. We underline the clinical importance of skin symptoms in the patients associated with cancer. Most important extramuscular manifestations in the CAM group were joint involvement and oesophagus involvement. Only 18.6% and 23.26% of patients with CAM had any MSAs or MAAs. All patients received steroids but other immunosuppressant drugs were not widely used in this group of patients.

Another interesting question is to explain what levels of muscle enzymes have occurred in our patients. In CAM patients, at onset of muscle symptoms, the mean value of creatine kinase levels was 3269.34 ± 4306.35 IU/L (2715.95 ± 3564.52 IU/L for DM and 4078.15 ± 5238 IU/L for PM patients), and the mean value of lactate dehydrogenase was 1019 ± 760.69 IU/L (1096.67 ± 752.07 IU/L for DM and 894.31 ± 788.16 IU/L for PM patients). In the control group, at onset of muscle symptoms, these mean values were 4543.16 ± 4542.09 IU/L for CK and 1248.58 ± 3222.55 IU/L for LDH (statistically significant higher than those in CAM patients: *p* values 0.048 and 0.021, respectively). CK and LDH of patients undergoing surgery were significantly reduced after intervention. CK just before surgery was 4297.20 ± 5584.24 IU/L and after surgery was 1144.43 ± 887.82 IU/L (*p* = 0.006); LDH right before surgery was 1126.38 ± 828.61 IU/L and after surgery was 653.19 ± 620.82 (*p* = 0.027).

We should also mention the oncological treatment of these 43 patients. In 24 cases (55.81%) surgical removal of the tumour occurred, 22 patients (51.1%) received chemotherapy, 17 patients (39.53%) received radiotherapy, 8 patients (18.6%) got hormone treatment, 5 extremely serious cases (11.62%) did not receive any anti-tumour treatment. A total of 22 patients (51.16%) received combination therapy (two, three or four types of treatment).

**Fig. 2:**
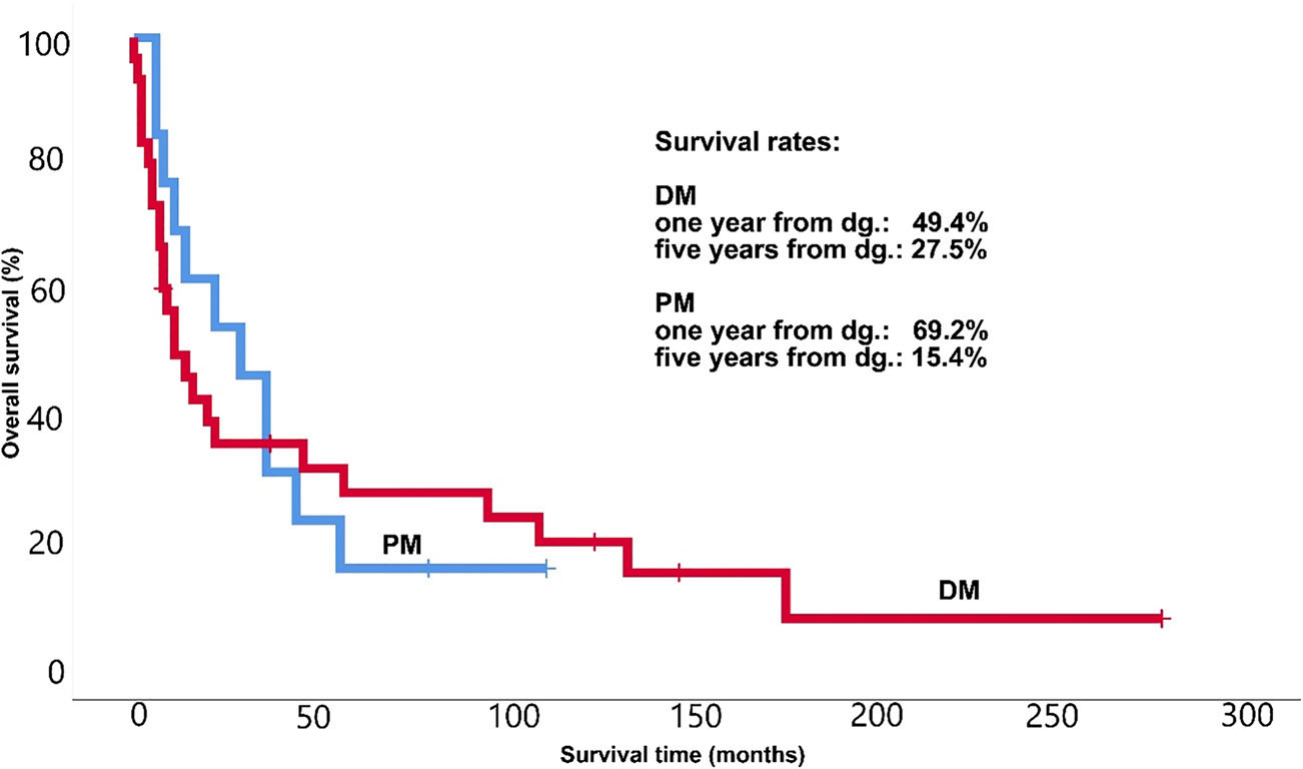
Long-lasting survival of our Hungarian DM and PM patients with malignancy

This question leads us to another important issue: survival of CAM patients. All together 36 of the 43 patients died (83.72%); 25 DM (83.33%) and 11 PM patients (84.62%). According to the statistical analysis the mean survival for the 43 CAM patients were 54.85 (29.44–80.26; 95% CI) months, the survival rates at one and at five years from diagnosis were 55.5% and 23.4%, respectively. These data for the 30 DM patients out of the 43 CAM patients are: mean survival 57.55 (25.06–89.04; 95% CI) months, the survival rates at one and at five years from diagnosis were 49.4% and 27.5%, respectively. The appropriate data for the 13 PM patients out of the 43 CAM patients are: mean survival 37.85 (18.83–56.86; 95% CI) months, the survival rates at one and at five years from diagnosis were 69.2% and only 15.4%, respectively. Survival rates can be followed on Fig. 2. Median survival of the 43 CAM patients were 16.00 (4.54–24.46; 95% CI) months. As mentioned earlier, the disease was extremely fulminant in five patients. In four cases 2–5 months after severe myositis symptoms, lung cancer was confirmed. Their survival was only 1–2 months after the diagnosis of the tumour; in their case, the cause of death was the following: respiratory failure due to respiratory muscle involvement; severe bronchitis; pneumonia; progression of the tumour process due to brain metastases. In another case, severe DM symptoms were associated with metastatic pancreatic cancer, and the patient died of liver failure. Cause of death of other CAM patients, apart from the above mentioned, were among others ileus, renal failure, tumour progression, metastases or sepsis.

## Discussion

Our department has decades of experience in the field of healing of patients with myositis. After 29 years of follow-up, we believe that it can be stated that cancer cases occurring two years before or three years after the diagnosis of myositis can be considered as CAM. In this study, more than 80% of the cancers were diagnosed within one year of onset of muscle symptoms which means that it is very important to think about the possibility of cancer at every single myositis patient. Out of the 450 myositis patients there were 43 CAM patients (9.56%); this means that one in every ten myositis patient is a CAM patient. In an Italian cohort it was 17% [23]. In our whole myositis population there were 30 DM-CAM (out of 146 patients; 20.55%) and 13 PM-CAM (out of 304 patients; 4.28%) patients. This reflects that: both DM and PM are associated with increased risk of malignancy but dermatomyositis is more associated with any type of cancer than polymyositis. This is similar to the results of other workgroups [24]. In a study from northern China, the frequency of malignancy in DM patients was 17.99%, similar as our result [25]. Some other interesting data of this Chinese cohort also should be discussed here. 69.77% of malignancy was diagnosed within the first year before or after the onset of myositis; this is a little bit lower than our result. The most frequent histology was adenocarcinoma but they found that lung cancer was the most frequent localisation. According to the most recent demographic data of the cancer registries (2006–2015) in Hungary the lung (males) and breast (females) cancers are at first place in cancer-morbidity hierarchy [26]. This is in harmony with our results. Just like us, other workgroups also detected other atypical types of tumour-localisation in CAM patients: ovarian cancer [27], colorectal cancer [28], haematological malignancy [29], cervical cancer [30], pancreatic tumour [31], carcinoma of the penis [32] or prostate cancer [33].

Comparing the CAM patients to the control group, as seen in Table 2., older age can be a risk factor for malignancy in myositis patients. 56.6 years at disease onset is the same as found by Sellami et al. in 2018 [34]. In our studied population there was no significant difference in female:male ratio. In contrast, a meta-analysis from China found that male sex increases the risk of malignancy [35]. As seen in Table 3., the frequency of ANF positivity in CAM patients was statistically significant lower than in patients without malignancy. The same result was concluded by Hoesly et al. in their study in 2018 [36]. Skin symptoms were more frequent in the CAM group this is because DM:PM ratio was significantly higher in these patients. There is data in the international literature that distal muscle weakness is more frequent in malignancy-associated cases; 16.28% of this group had this kind of muscle symptom and this is a significant difference between the two groups. Perhaps it also can be a warning sign for cancer. The frequency of Raynaud’s phenomenon was significantly lower in the malignancy-related group. In a high percentage of cases it was asymmetric and that can be the first sign of malignancy [37]. There was no significant difference between the two populations in the frequency of extramuscular manifestations (arthralgia, dysphagia, heart involvement and lung fibrosis). A meta-analysis found that malignancy was associated with a reduced risk of developing ILD [38]. Another systemic review said that several factors were associated with lower risk of malignancy, including the presence of ILD, arthritis/arthralgia or anti-Jo-1 antibody [39]. As seen in Table 3., we cannot strengthen these data. But Lu et al., as well as our workgroup, found that the Raynaud’s phenomenon is associated with lower-than-average risk for malignancy [39].

Despite anti-tumour therapy, survival rate of our CAM patients was very poor. The survival rate of primary PM and DM group at 5 years was significantly higher (100% and 100%), compared with the CAM-patient group, where survival was 15.4% for PM and 27.5% for DM. Although the risk in PM patients is lower to have cancer, there is less chance of long-lasting survival. Neri et al. found that the chance of long-lasting survival is higher in PM patients [23]. Regarding anti-myositis therapy immunosuppressant drugs were less frequently used in CAM patients than in simple myositis patients. Despite the widely studied association between myositis and cancer the best strategy for diagnosing and treat cancer in IIM patients is lacking [40].

## Conclusion

Authors underline the clinical importance of the fast diagnosis and the best possible therapy in patients with cancer-associated myositis. According to the results of this retrospective review the risk of malignancy is present in both genders and higher age groups and is highest in the first year before or after onset of myositis. Patients with DM have a higher incidence of malignancy than patients with PM and both groups have a poor prognosis. Adults should be evaluated for malignancy at diagnosis, followed by long-term surveillance.
